# Feasibility of Cowpea chlorotic mottle virus-like particles as scaffold for epitope presentations

**DOI:** 10.1186/s12896-015-0180-6

**Published:** 2015-08-27

**Authors:** Afshin Hassani-Mehraban, Sjoerd Creutzburg, Luc van Heereveld, Richard Kormelink

**Affiliations:** Laboratory of Virology, Wageningen University, Droevendaalsesteeg 1, 6708 PB Wageningen, The Netherlands

**Keywords:** CCMV, viral nanoparticles, virus-like particles, VLP, Influenza A virus, Foot and Mouth Disease Virus, Schmallenberg virus, prokaryote expression system

## Abstract

**Background & Methods:**

Within the last decade Virus-Like Particles (VLPs) have increasingly received attention from scientists for their use as a carrier of (peptide) molecules or as scaffold to present epitopes for use in subunit vaccines. To test the feasibility of *Cowpea chlorotic mottle virus* (CCMV) particles as a scaffold for epitope presentation and identify sites for epitope fusion or insertion that would not interfere with virus-like-particle formation, chimeric CCMV coat protein (CP) gene constructs were engineered, followed by expression in *E. coli* and assessment of VLP formation. Various constructs were made encoding a 6x-His-tag, or selected epitopes from Influenza A virus [IAV] (M2e, HA) or Foot and Mouth Disease Virus [FMDV] (VP1 and 2C). The epitopes were either inserted 1) in predicted exposed loop structures of the CCMV CP protein, 2) fused to the amino- (N) or carboxyl-terminal (C) ends, or 3) to a N-terminal 24 amino acid (aa) deletion mutant (N∆24-CP) of the CP protein.

**Results:**

High levels of insoluble protein expression, relative to proteins from the entire cell lysate, were obtained for CCMV CP and all chimeric derivatives. A straightforward protocol was used that, without the use of purification columns, successfully enabled CCMV CP protein solubilization, reassembly and subsequent collection of CCMV CP VLPs. While insertions of His-tag or M2e (7-23 aa) into the predicted external loop structures did abolish VLP formation, high yields of VLPs were obtained with all fusions of His-tag or various epitopes (13- 27 aa) from IAV and FMDV at the N- or C-terminal ends of CCMV CP or N∆24-CP. VLPs derived from CCMV CP still encapsulated RNA, while those from CCMV CP-chimera containing a negatively charged N-terminal domain had lost this ability. The usefulness and rapid ease of exploitation of CCMV VLPs for the production of potential subunit vaccines was demonstrated with the synthesis of chimeric CCMV VLPs containing selected sequences from the G_N_ and G_C_ glycoproteins of the recently emerged Schmallenberg orthobunyavirus at both termini of the CP protein.

**Conclusions:**

CCMV VLPs can be successfully exploited as scaffold for epitope fusions up to 31 aa at the N- and C-terminus, and at a N-terminal 24 amino acid (aa) deletion mutant (N∆24-CP) of the CP protein.

## Backgound

Virus-like particles (VLPs) have become recognized as ideal biopolymers for various applications within Bionanotechnology [1], amongst which the production of vaccines. Among the various types of vaccines produced, subunit vaccines receive a particular interest, because only small antigenic parts of a target pathogen are being used for immunization. This requires selection of an immuno-dominant peptide that on its own still induces a strong immune response capable to (partially) protect against invasion with the pathogen from which the peptide was derived. A major advantage of a subunit vaccine is the lack of any risk, i.e. the chance that the vaccine might elicit a counter response, due to the absence of the pathogen [2]. Furthermore, a subunit vaccine can in principle be produced in many different protein expression systems. However, a drawback of subunit vaccines is the requirement for multiple doses and the use of efficient but nontoxic adjuvant formulations to confer acceptable protection. It is often due to poor immunogenicity that has limited the application of these vaccines [3]. This is most likely caused by improper folding and/or poor presentation of the antigen subunits to the immune system [4]. To enhance the immunogenic properties, antigenic subunits are being offered with stronger adjuvants [5] or more and more displayed on a carrier molecule like VLPs. Besides being non-infectious, due to the lack of the viral genome, VLPs resemble authentic viral particles in their unique structure (and size) and thereby also retain their ability to induce a strong B cell response, i.e. elicit neutralizing antibodies in the absence of adjuvants [6–8]. Meanwhile, epitope based-VLP vaccines have been produced in which only a small antigenic stretch(es) of a target protein(s) has been incorporated into VLPs and shown to strongly induce innate and cognate immune responses and enhancing the generation of antibodies without application of adjuvants [7–9].

To date a variety of plant and animal VLPs have been developed and exploited for peptide/epitope display by genetic fusion or chemical conjugation [6] which include Hepatitis B core antigen [10], Papilloma VLPs [11], Bacteriophage Qβ [12] and Papaya mosaic VLPs [13, 14]. Ideal VLP candidates contain at least one tolerable site for the fusion or insertion of foreign epitopes without any side effects or toxicity to the recipient [15–17]. VLPs derived from plant viruses have the additional advantage that vertebrate hosts have never faced an infection with these pathogens; hence will not neutralize these VLPs once they are being used for immunization. Thus far several plant viruses have been studied and a few have been tested in animal models for their potential vaccine (Table 1). Among plant viruses, CCMV has not been extensively studied for these purposes yet, although CCMV VLPs are reported and have some unique and interesting features. CCMV is a single-stranded (+)-sense RNA virus and member of the *Bromovirus* genus within the family of *Bromoviridae*. It consists of spherical particles, approximately 28 nm in diameter and capsid entirely composed of a single coat protein (CP) of about 20 kDa [18]. The icosahedral particle (*T = 3*) symmetry is composed of 180 copies of the CP subunit assembled into 12 pentamers and 20 hexamers. The CP protein consists of 190 aa residues and is composed of an eight β-barrel core (residues 52-176) from which the amino- (N) (residues 1-51) and carboxyl- (C) (residues 176-190) termini extend in opposite directions [19]. The N terminus is highly positively charged and the first 26 aa project into the capsid interior and contribute to an electrostatic interaction with viral RNA [20]. The C-terminus lies tangentially to the capsid structure, wedging between the N-terminus and β-barrel structure of the neighbouring CP subunits, and stabilizes dimeric interactions [21].

**Table 1:** Plant virus-like particles used as scaffolds for epitope presentation

Virus	Particle shape	Candidate epitope	Reference
Potato leafroll virus (PLRV)	Spherical	His	[44]
Tomato bushy stunt virus (TBSV)	Spherical	V3 & RTA	[45, 46]
Tobacco mosaic virus (TMV)	Rod	HVR1	[47]
Cowpea mosaic virus (CPMV)	Spherical	Nlm-1A	[48]
Johnson grass mosaic virus (JGMV)	Filamentous	Peptide A,B,C,D	[49]
Alfalfa mosaic virus (AMV)	Bacilliform	PA-D4s	[50]
Physalis mottle virus (PhMV)	Spherical	3B1, 3B2, 3AB, 3D,3ABD	[51]
Cucumber mosaic virus (CMV)	Spherical	F & HN	[52]
Papaya mosaic virus (PapMV)	Filamentous	p33-CTL, M2e & HA11	[13, 14, 53]
Cymbidium ringspot virus (CymRSV)	Spherical	Myc	[54]
Potato virus Y (PVY)	Filamentous	preS1	[55]
Bamboo mosaic virus (BaMV)	Filamentous	VP2	[56]
Turnip mosaic virus (TuMV)	Filamentous	VEGFR-3	[57]
Cardamom mosaic virus (CdMV)	Filamentous	gp41	[58]
Artichoke mottled crinkle virus (AMCV)	Spherical	2 F5	[59]

One of the highly interesting features of CCMV particles is that these are able to self-assemble *in vitro* in a reversible manner, depending on pH, ionic conditions and concentration of divalent cations (Ca^2+^, Mg^2+^). At pH above 7.0 CCMV particles disassemble while lowering the pH below 6, low ionic strength (*i* = 0.2) [19] and the additional presence of divalent cations (0.01 M) support reassembly of CCMV VLPs. When such process is performed in a subtle manner, CCMV VLPs can be manipulated to only swell without disassembly and thereby allow encapsulation of small molecules into the core of the particle. In this respect, CCMV VLPs have been exploited as nanocarriers to encapsulate different types of cargo molecules i.e. proteins, fluorophore and/or metal ions [22, 23].

CCMV VLPs have been expressed in several heterologous expression systems i.e. yeast [24], insect cell (unpublished observation), prokaryotic [19] and analyzed for VLP formation. High yield of CCMV VLPs and soluble assembled VLPs were only obtained in yeast (*Pichia pastoris*) [25] and *Pseudomonas fluorescens* [26]. A recent study furthermore showed that wild type CCMV VLPs generally have a uniform distribution and do not show overt toxicity in naïve and immunized mouse which makes CCMV nanoparticles very attractive for biomedical applications in terms of their safety and biocompatibility [24].

Studies on CCMV-based VLPs as an epitope presentation system, however, are still limited and the information as to where fusions and insertions of smaller and/or larger epitopes can be made to exploit CCMV VLPs as scaffold for vaccine purposes is not available.

Thus far, only the highly conserved ectodomain of matrix 2 protein (M2e) of Influenza A virus has been cross-linked to the surface of CCMV VLPs, or fused to the N terminus and shown to maintain VLP formation [27]. Here we present a more extensive study in which we analyzed four predicted loop structures within CCMV CP [22] and the N- and C-termini for their potential use as target sites to insert/fuse epitopes whilst maintaining VLP formation.

## Results

### Selection of putative epitope insertion and fusion sites

**Fig. 1:**
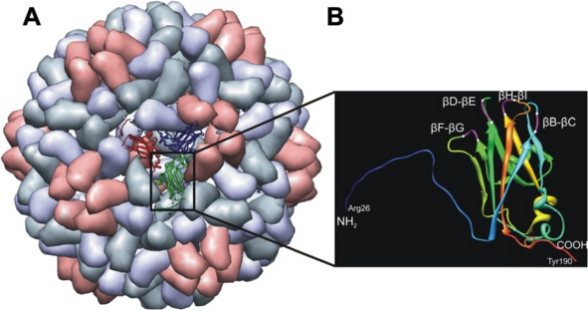
Schematical presentation of a CCMV virion and a coat protein subunit. **a** CCMV particle according to data from Protein Data Bank (PDB ID: 1ZA7 [http://www.rcsb.org/pdb/home/home.do]) [20] and as visualized by Chimera1.6.2 (http://www.cgl.ucsf.edu/chimera/) [60], showing an icosahedral asymmetric unit consisting of three identical subunits in the centre, **b** ribbon diagram of a coat protein subunit B displaying N-terminal end (residues 1-25 are not shown), four β-barrels (βB-βC, βD-βE, βF-βG and βH-βI) and C-terminal end as potential insertion sites. Insertions within the barrels are shown between the white dashes

**Fig. 2:**
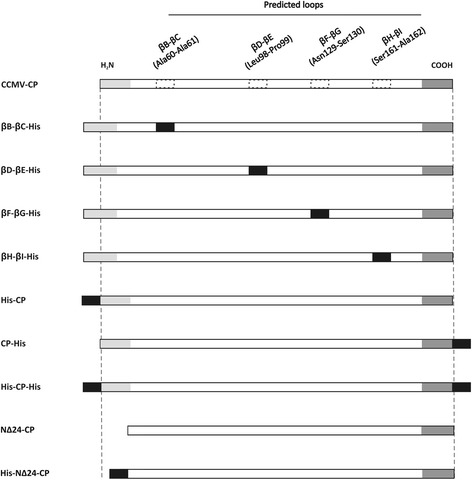
Schematical diagram of CCMV-CP and NΔ24-CP constructs expressed in *E. coli*. Six His-tag residues introduced within the predicted loops (βB-βC-His, βD-βE-His, βF-βG-His and βH-βI-His) were cloned as *Spe*I-*Sst*I, while for the N- and C-terminal end fusion of the CCMV-CP (His-CP, CP-His and His-CP-His) and NΔ24-CP (His-NΔ24-CP and His-HA-NΔ24-CP), the 6xHis-tags were introduced by PCR

Potential sites to be tested for fusion and external exposures of epitopes on CCMV VLPs were selected based on analysis of the CCMV CP crystal structure (Fig. 1). Based on this, four predicted loops, named βB-βC, βD-βE, βF-βG- and βH-βI, and the N- and C-termini were selected for insertions respectively fusions (Fig. 2).

### Expression and VLP formation of chimeric His-tagged CCMV CP and NΔ24-CP

**Fig. 3:**
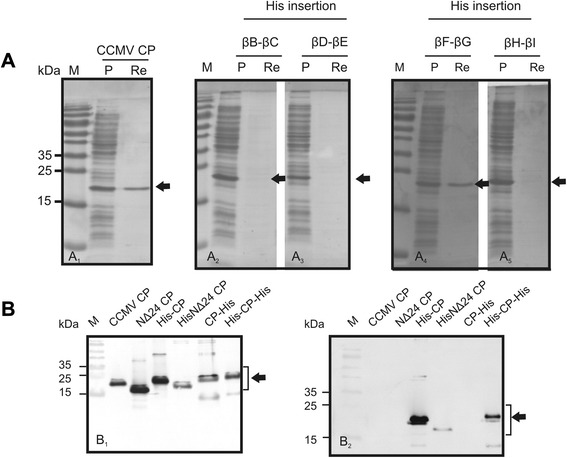
Expression of His-tag insertion and terminal fusion CCMV constructs in *E. coli*. **a** Cell extracts (CE) and reassembled VLP pellets (RP) collected through sucrose cushion of CCMV-CP (*A*_1_) and CCMP-CP derivatives containing a 6xHis-tag insertion within each of the loop structures (βB-βC-His [*A*_2_], βD-βE-His[*A*_3_], βF-βG-His[*A*_4_] and βH-βI-His[*A*_5_]), were resolved on 15 % SDS-PAGE and stained with Coomassie blue. **b** Western blots of 6xHis-tag fusion constructs (His-CP, CP-His, His-CP-His) detected by polyclonal anti-CCMV (*B*_1_) and monoclonal anti-His (*B*_2_) sera. CCMV-CP and NΔ24-CP were used as controls for size comparison with the chimeras. A molecular size marker is indicated on the left

**Fig. 4:**
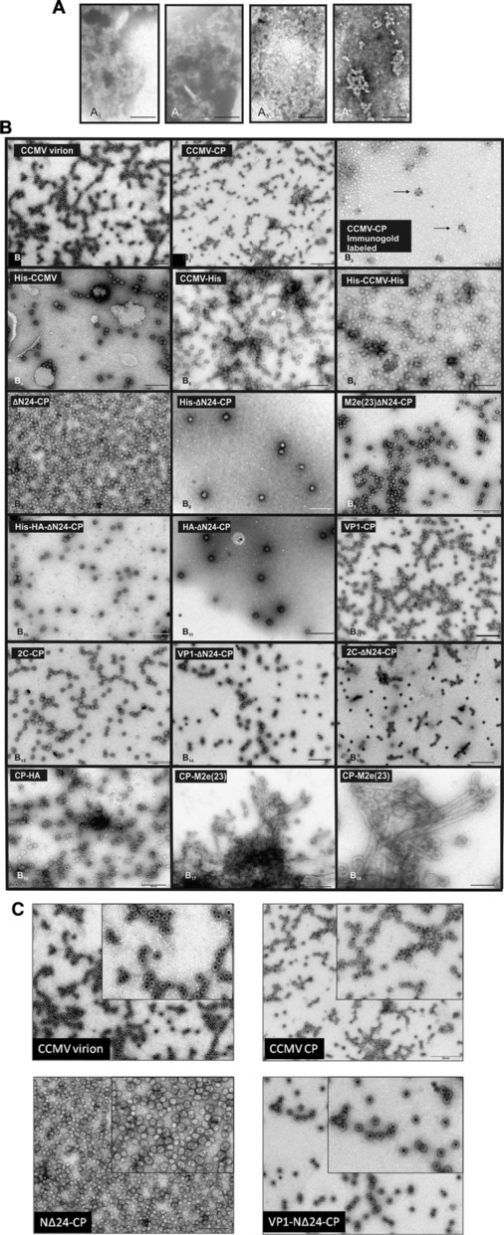
Electron micrographs of negatively-stained CCMV-CP/ NΔ24-CP loop insertion and fusion constructs expressed in *E. coli*. (**a**) Reassembled VLP pellet of βF-βG-His (A_1_), βF-βG-M2e(7) (A_2_), βF-βG-M2e(15) (A_3_) and βH-βI-M2e(23) (A_4_). (**b**) CCMV virions purified from cowpea plants (B_1_), CCMV-CP VLPs (B_2_), immuno-gold labeled CCMV-CP (B_3_), His-CCMV (B_4_), CCMV-His (B_5_), His-CCMV-His (B_6_), NΔ24-CP (B_7_), His-NΔ24-CP (B_8_), M2e(23)NΔ24-CP (B_9_), His-HA-NΔ24-CP (B_10_), HA-NΔ24-CP (B_11_), VP1-CP (B_12_), 2C-CP (B_13_), VP1-NΔ24-CP (B_14_), 2C-NΔ24-CP (B_15_), CP-HA (B_16_), CP-M2e(23) (B_17_, B_18_).(**c**) Higher resolution of four selected CCMV particles including CCMV virion, CCMV-CP, NΔ24-CP and VP1-NΔ24-CP shown on the top-right of each electron micrograph. Magnification 20,000x, scale bar represents 200 nm

To test whether small heterologous sequences could be inserted or fused, without abrogation of CCMV VLP formation, a first series of CCMV CP chimeras was made in which a 6xHis-tag was inserted in any of the four selected and predicted loops, and fused to the N- or C-terminus (Fig. 2). In addition a CP construct was generated lacking the first 24 aa (denoted NΔ24-CP), to be tested on the ability to still fold into VLPs and potential future exploitation. Constructs were cloned into pET 28a (Novagen) expression vector, expressed in BL21 cells and subsequently analyzed on sodium dodecylsulfate-polyacrylamide gels. Polyacrylamide gels were stained with Coomassie brilliant blue and showed the presence of protein bands that were absent from BL21 control cells, and corresponded with the expected sizes of the CCMV CP chimera and NΔ24-CP. Further analysis revealed that most of these chimeric proteins ended up in the insoluble fraction (Fig. 3 and unpublished observation). For this reason, a protocol was developed to solubilize chimeric CP proteins from protein aggregates and *in vitro* reassemble them into VLPs. In a final step highly purified VLPs were collected from a pellet after ultracentrifugation on 30 % sucrose cushion, as consistently observed when applied on wt CCMV CP protein (Fig. 3a_1_, b). Following this procedure, none of the CCMV CP proteins containing His-tag insertions in the loops were found ending up in the pellet fraction except for the CP βF-βG-His protein (Fig. 3a, panels a_2-4_). On the other hand, expressed proteins were obtained at concentration range of 36-63 mg/l in pellets prepared from lysates expressing wt CCMV CP and CP proteins containing a His-tag at the N- or C-terminus of CCMV-CP or at the N-terminus of NΔ24-CP (Fig. 3b). Since detection of wt CCMV CP protein in these pellets consistently correlated with the presence of VLPs, the presence of pellets after sucrose cushion ultracentrifugation for the other CCMV CP chimeric proteins was indicative for the formation of VLPs. Similar to CCMV CP, EM analysis from resuspended pellet material indeed confirmed the formation and presence of VLPs for CCMV CP-chimeric proteins containing a single 6xHis-tag at its N- or C-terminus and also when present at both termini simultaneously (Fig. 4b_1-6_). For the CCMV CP chimera containing a 6xHis-tag in the βF-βG loop, and for which small amounts of macro molecular protein complexes (MMPCs) were found in the pellets after sucrose gradients, no VLPs were observed. Instead, large aggregates were observed (Fig. 4a_1_). Pellets obtained from NΔ24-CP also revealed the presence of VLPs (Fig. 4b_7_).

Whereas the CCMV CP construct containing the C-terminal 6xHis-tag became well expressed (Fig. 3b_1_), and the presence of the 6xHis-tag encoding nucleotide sequence was verified by nucleotide sequence analyses, the His-tag could not be detected on western immunoblots using anti-His serum (Fig. 3b_2_).

### Expression and VLP formation of CCMV CP containing IAV M2e epitope insertions

**Fig. 5:**
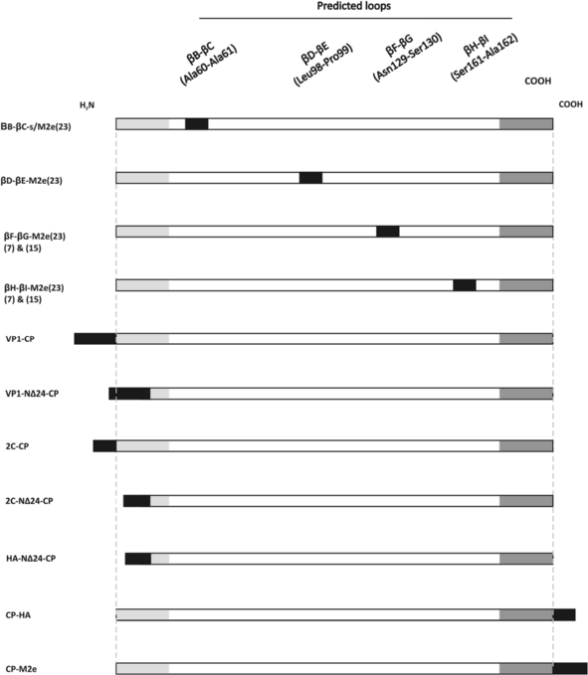
Schematic illustration of loop-inserted and coat protein fusion constructs expressed in *E. coli*. The ectodomain of M2e-protein of Influenza A virus: M2e(23), M2e(15) and M2e(7) introduced within the predicted loops (βB-βC-M2e(23), βD-βE-M2e(23), βF-βG-M2e(23)/M2e(15)/M2e(7) and βH-βI-(23)/M2e(15)/M2e(7) as adaptors via *Spe*I and *Sst*I overhangs. Foot and mouth disease virus (VP1/2C) Influenza A virus epitopes [M2e(23) and HA] were fused by PCR to the N-terminus of the CCMV-CP (VP1/2C-CP) and NΔ24-CP (VP1/2C/M2e/HA-NΔ24-CP), and C-terminal to CCMV-CP (CP-HA, CP-M2e)

**Fig. 6:**
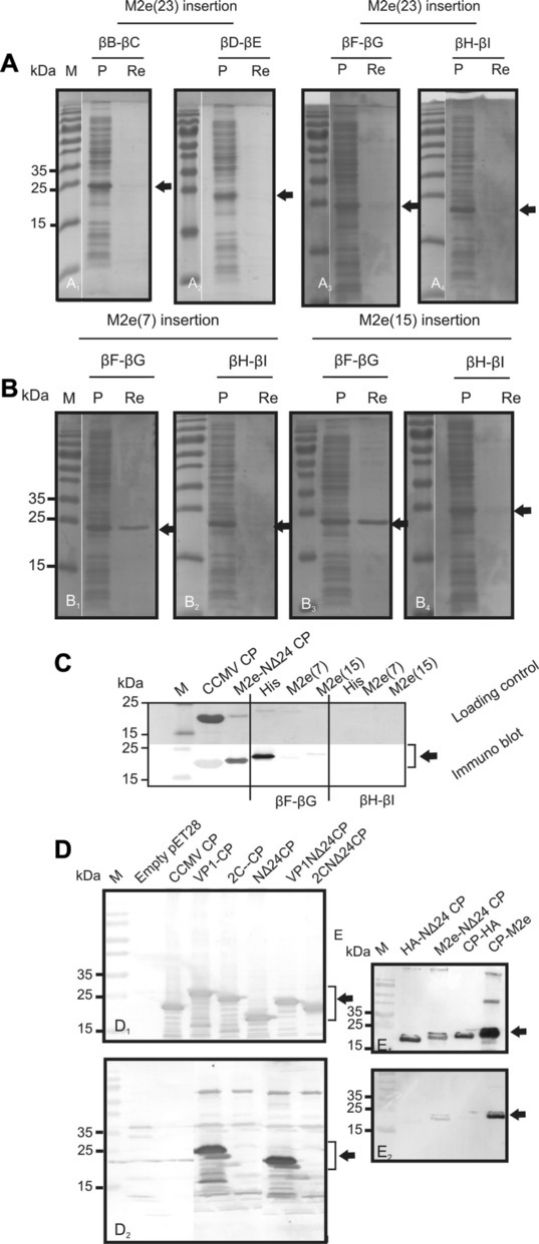
Expression of loop-inserted and terminal fusion CCMV constructs in *E. coli*. (**a**) SDS-PAGE analysis of cell extract (CE) and reassembled VLP pellet (RP) from the M2e(23) loop-inserted CCMV-CP (A_1_) βB-βC-M2e(23), (A_2_) βD-βE-M2e(23), (A_3_) βF-βG-M2e(23) and (A_4_) βH-βI-M2e(23). (**b**) SDS-PAGE analysis of M2e(7) and M2e(15) within βF-βG (B_1_,B_3_) and βH-βI(B_2_, B_4_) CCMV-CP loops. (**c**) SDS-PAGE (upper panel) and western blot (lower panel) of βF-βG M2e(7)/M2e(15) and βH-βI M2e(7)/M2e(15) detected by monoclonal anti-His-tag and M2e sera, respectively. (**d**) Immunoblot detection of VP1 and 2C fusions to CCMV-CP and NΔ24-CP using polyclonal anti-CCMV serum (D_1_) and serum from FMDV-infected guinea pig (D_2_). Induced empty pET-28a and CCMV-CP were used as negative and positive control, respectively. (**e**) Detection of M2e fused to NΔ24CP using polyclonal anti-CCMV (E_1_) and monoclonal anti-M2e (E_2_) sera. Molecular size marker is indicated on the left

To rule out that abrogation of VLP formation in case of insertion into the loops was not just due to the 6xHis-tag, a similar set of insertional CCMV CP chimeras was made but instead of a His-tag now containing the 23 aa IAV M2e epitope (Fig. 5). Constructs made were cloned into pET28 and verified by sequence analysis, and after expression in *E.coli* further processed for *in vitro* VLP reassembly and purification by ultracentrifugation. Whereas all constructs of CCMV CP containing the M2e epitope insertion into one of the loops became well expressed (Fig. 6a), only very low amounts of the βF-βG-M2e(23) and βH-βI-M2e(23) proteins (Fig. 6a_3_ and a_4_) could be observed in the pellets after sucrose cushion. To test whether these results were influenced by the size of the M2e (23 aa) insertion, a range of constructs containing smaller insertions (7 or 15 aa) were made and analyzed (Fig. 5). While proteins from the insertional CCMV chimera βF-βG-M2e(7) and -M2e(15) were detected in pellets after sucrose cushion (Fig. 6b_1_ and b_3_), only very low amounts were detected for those of βH-βI-M2e(7) and -M2e(15) (Fig. 6b_2_ and b_4_). Western immunoblot analysis of all CCMV βF-βG loop-insertions showed a good detection of the His-tag but a weaker one of the M2e(7) and M2e(15) insertions, likely due to lower reactivity of the antiserum to a smaller M2e sequence insertion (Fig. 6c). Although the presence of these proteins in pellets obtained after sucrose cushion ultracentrifugation were indicative for MMPC, and consistently observed to correlate with VLP formation of wild type CCMV CP (Fig. 4b_1-3_), none of the βF-βG loop CCMV CP chimera for which also MMPCs were found in the pellets, did fold into VLPs. Instead, large and similar aggregates were observed for all βF-βG loop chimera, as shown for βF-βG-His, βF-βG-M2e(7), βF-βG-M2e(15) and βF-βG-M2e(23)( Fig. 4a_1-4_).

### Expression and VLP formation of CCMV CP containing IAV and FMDV epitope N- and C-terminal fusions

To further substantiate the observation that N- and C-terminal fusions, in contrast to insertions, did not abolish the reassembly of CCMV CP into VLPs, additional CCMV CP chimera were tested containing different epitopes derived from FMDV (VP1, 2C) and IAV (M2e, HA) and varying in size, at the N- or C-terminus of CCMV CP or at the N-terminus of the NΔ24 deletion mutant (See Methods and Fig. 5).

Clones containing FMDV and IAV epitope sequences were selected and after being confirmed by sequence analysis, were expressed in *E. coli* and analyzed for protein expression. Upon SDS-PAGE and Western immunoblot analysis using anti CCMV CP serum, all CCMV CP chimeras containing either IAV or FMDV epitopes were well detected using anti CCMV CP serum (Fig. 6d_1_ and e_1_). Chimeras containing VP1 or M2e fusion proteins additionally were detected with serum from FMDV-infected animals and anti-M2e serum, respectively (Fig. 6d_2_ and E_2_), confirming the presence of those epitopes. CCMV CP chimera containing FMDV 2C fusions did not react with the FMDV antiserum. Since the presence of 2C and HA epitopes was not confirmed serologically (Fig. 6d_2_), their presence relied on the confirmation by sequence analysis of the constructs and the observation that the size of the chimeric proteins detected using anti-CCMV serum corresponded to the expected size of the predicted protein.

After protein samples were subjected to VLP reassembly and sucrose cushion ultracentrifugation, pellets obtained were resuspended and analyzed by EM. Similar to the results earlier obtained with His-tag fusions at the N- or C-termini of CCMV CP, an N-terminal His-tag fusion to NΔ24-CP did not abolish VLP formation (Fig. 4b_8_). N-terminal fusions of M2e(23), HA, VP1 and 2C to CCMV CP or NΔ24-CP all resulted in the formation of VLPs (Fig. 4b_9_–b_17_). When His and HA were combined as a fusion at the N-terminus of CCMV CP, VLPs were also observed (Fig. 4b_10_). While fusion of HA at the C-terminus of CCMV CP, similar to the earlier observed 6xHis-tag fusion, still maintained VLP formation, fusions with M2e(23aa) abolished VLP formation. Instead, tubular-like structures were observed (Fig. 4b_16_–b_18_). During VLP reassembly and EM analysis, purified CCMV virions and CCMV CP were included as positive controls (Fig. 4b: b_1-_b_3_). Higher resolution of 4 selected VLP samples are shown as examples for CCMV VLP identification and to provide a more detailed view on the structure of the VLPs generated (Fig. 4c).

**Fig. 7:**
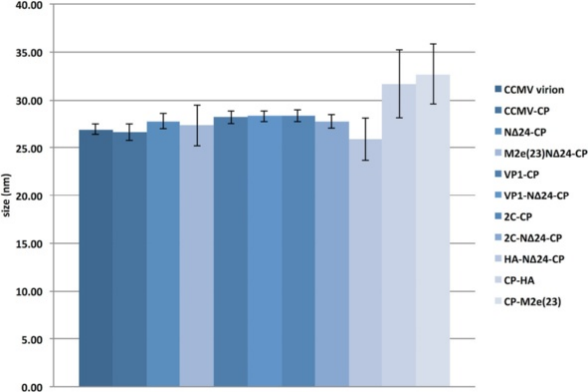
Size measurement of chimeric CCMV-CP/ NΔ24-CP VLPs. The average size of VLPs from CCMV-CP, NΔ24-CP, M2e(23)NΔ24-CP, VP1-CP, VP1-NΔ24-CP, 2C-CP, 2C-NΔ24-CP, HA-NΔ24-CP, CP-HA and CP-M2e(23) are presented in comparison to the size of plant-purified CCMV virions

To find out whether epitopes fused at the N or C-terminus were exposed on the outside of VLPs, immunogold electron microscopy was performed on CCMV VLPs containing N-terminal and C-terminal His- or M2e-fusions. However, during several attempts no immunogold labeling was observed, indicated a possible internalization of the fusions. While the number of VLPs observed varied between the different CCMV CP chimera, most VLPs consistently revealed an average size ranging between 26.8 nm and 28.3 nm. Only those for CP-HA and CP-M2e(23) on average were larger, i.e. 31.7 ± 3.6 nm respectively 32.7 ± 3.1 nm (Fig. 7).

### Encapsulation of mRNA

**Fig. 8:**
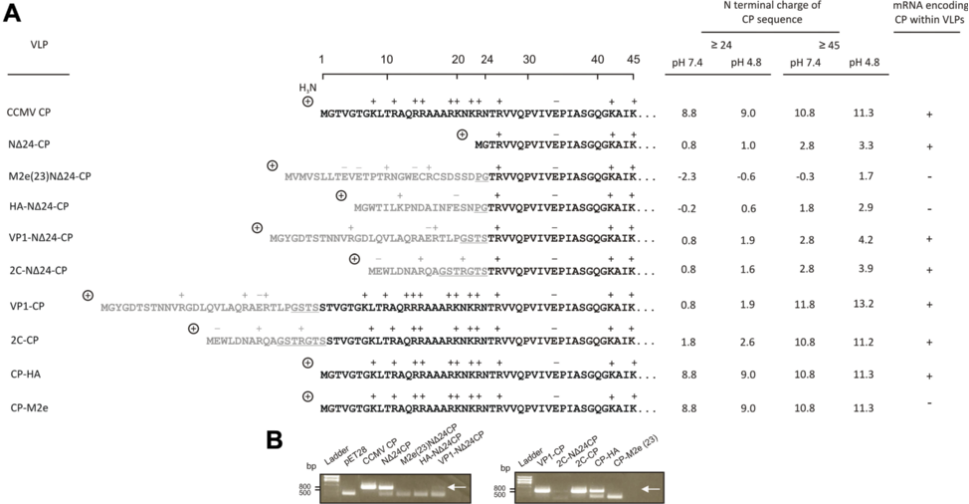
N-terminal charge of chimeric subunits from VLPs and detection of mRNAs. **a** N-terminal charges of CCMV-CP, NΔ24-CP, M2e(23)NΔ24-CP, VP1-CP, VP1-NΔ24-CP, 2C-CP, 2C-NΔ24-CP, HA-NΔ24-CP, CP-HA and CP-M2e(23) were calculated at pH 7.2 and 4.8 for ≥24 and ≥45 N- terminal residues using Protein Calculator version 3.3 (http://www.scripps.edu/~cdputnam/protcalc.html). **b** RT-PCR detection of mRNAs encoding CCMV coat proteins. ⊕Positive charge derived from NH_2_ group. (+) indicates the presence of RNA followed by PCR product cloning and sequence confirmation and (-) for its absence not being cloned and sequenced

Since the N-terminal domain of CCMV CP is positively charged (Fig. 8a) and involved in the interaction and encapsulation of (viral) RNA [19], the presence of (m) RNA within CCMV CP-derived VLPs, through sequence non-specific interactions, was anticipated. To test this, VLPs from CCMV-CP chimera were subjected to a total RNA purification protocol and the RNA collected analyzed by RT-PCR for the presence of RNA. Due to the known sequence (bacterially synthesized) CCMV CP transcripts were selected for amplification, using specific primers for the CP gene (Fig. 8b and Table 2). VLPs derived from CCMV CP chimera that lacked the first 24 aa (NΔ24-CP) were included. While the samples of CCMV-CP, NΔ24-CP, VP1-CP, 2C-CP and CP-HA gave a clear positive RT-PCR product of expected size, those from VP1-NΔ24CP and 2C- NΔ24CP only gave a very weak signal. PCR products from clear positive samples were cloned and sequenced and confirmed the presence of NΔ24CP/CCMV-CP mRNA in VLPs. In contrast, no RNA was detected at all in VLP samples from M2e(23)NΔ24-CP, HA-NΔ24-CP and CP-M2e (Fig. 8b). To find out whether encapsulation of RNA could be correlated to epitope sequences with a positive charge, similar to the original N-terminus of CP, the charges of the N-terminal domain from the CCMV CP-derived chimera were calculated (see Materials and Methods) at pH 7.4 and 4.8, corresponding to buffer conditions reflecting the CCMV CP disassembly and CCMV VLP reassembly steps. Only two out of eight proteins, i.e. M2e(23)NΔ24-CP and HA-NΔ24-CP and from which the VLPs did not encapsulate RNA, showed a negative charge at pH 7.4, whereas all other proteins revealed a range of positive charges. Furthermore, only M2e(23)NΔ24-CP revealed negative charge at pH 4.8.

**Table 2:** Primer and adapter sequences used in this study

Construct	Primer set 5ˊ → 3ˊ
CCMV-CP	GCGCCATGGGTACAGTCGGAACAGGGAAGTTAACTC (F)
	GGAAGCTTCACTAATACACCGGAGTG (R)
NΔ24CP	GGGCCATGGGGACTCGTGTGGTCCAAC (F)
	GGAAGCTTCACTAATACACCGGAGTG (R)
His-LBC	CGCACTAGTCATCACCATCACCATCACGAGCTCGCTGCCGAAGCTAAAGTAACCTCG (F)
	CGCACTAGTCGCACAAGAGGCGGTCCAC (R)
His-LDE	CGCACTAGTCATCACCATCACCATCACGAGCTCCCCAGTGTTAGTGGCACAGTGAAATC (F)
	CGCACTAGTAAGCAACCCAAGCCATAATAAAACTCTACC (R)
His-LFG	CGCACTAGTCATCACCATCACCATCACGAGCTCTCGAAAGATGTTGTCGCTGCTATGTAC (F)
	CGCACTAGTGTTGTCGGCCACAGCTAATGCC (R)
His-LHI	CGCACTAGTCATCACCATCACCATCACGAGCTCGCGGCTCTCACTGAGGGCGAC (F)
	CGCACTAGTACTGCTGTACAAGTAGATCGTTAAATCCGC (R)
M2e (7) loop adaptor	CTAGTCTGCTGACCGAAGTGGAGCT (F)
	CCACTTCGGTCAGCAGA (R)
M2e (15) loop adaptor	CTAGTCTGCTGACCGAAGTGGAAACCCCGACCCGCAACGGCTGGGAGCT (F)
	CCCAGCCGTTGCGGGTCGGGGTTTCCACTTCGGTCAGCAGA (R)
M2e (23) loop adaptor	CTAGTAGCCTGCTGACCGAAGTGGAAACCCCGACCCGCAACGGCTGGGAATGCCGCTGCAGCGAT
	AGCAGCGATGAGCT (F)
	CATCGCTGCTATCGCTGCAGCGGCATTCCCAGCCGTTGCGGGTCGGGGTTTCCACTTCGGTCAGCA
	GGCTA (R)
His-CP*	GGGCCATGGGTCACCACCACCACCACCACACAGTCGGAACAGGGAAGTTAAC (F)
	CCCAAGCTTCACTAATACACCGGAGTGAAAGAGTCGTCAAACGTAGGTCTGAC (R)
CP-His*	CGCCCATGGGTACAGTCGGAACAGGGAAGTTAACTCGTGCACAACGAAGGGC (F)
	GGGAAGCTTCACTAGTGGTGGTGGTGGTGGTGATACACCGGAGTGAAAGAGTCGTC (R)
His-CP-His*	GGGCCATGGGTCACCACCACCACCACCACACAGTCGGAACAGGGAAGTTAAC (F)
	GGGAAGCTTCACTAGTGGTGGTGGTGGTGGTGATACACCGGAGTGAAAGAGTCGTC (R)
His- NΔ24CP *	GGGCCATGGGTCACCACCACCACCACCACACTCGTGTGGTCCAACC (F)
	CCCAAGCTTCACTAATACACCGGAGTGAAAGAGTCGTCAAACGTAGGTCTGAC (R)
HA- NΔ24CP*	GGGCCATGGGGTGGACCATTCTGAAACCGAACGATGCGATTAACTTTGAAAGCAACCCCGGGACTCGTGTGGTCCAACCTG (F)
	CCCAAGCTTCACTAATACACCGGAGTGAAAGAGTCGTCAAACGTAGGTCTGAC (R)
His-HA- NΔ24CP*	GGGCCATGGGGCACCACCACCACCACCACTGGACCATTCTGAAACCGAAC (F)
	CCCAAGCTTCACTAATACACCGGAGTGAAAGAGTCGTCAAACGTAGGTCTGAC (R)
M2e(23) NΔ24CP*	CAACGGCTGGGAATGCCGCTGCAGCGATAGCAGCGATCCCGGGACTCGTGTGGTCCAACCTG (F1)
	GGGCCATGGTCATGGTCAGCCTGCTGACCGAAGTGGAAACCCCGACCCGCAACGGCTGGGAATGCCGCTGC (F2)
	CCCAAGCTTCACTAATACACCGGAGTGAAAGAGTCGTCAAACGTAGGTCTGAC (R)
CP-HA*	CGCCCATGGGTACAGTCGGAACAGGGAAGTTAACTCGTGCACAACGAAGGGC (F)
	CGTTCGGTTTCAGAATGGTCCAATACACCGGAGTGAAAGAGTCG (R1)
	CCCAAGCTTCACTAGTTGCTTTCAAAGTTAATCGCATCGTTCGGTTTCAGAATGG (R2)
CP-M2e(23)*	CGCCCATGGGTACAGTCGGAACAGGGAAGTTAACTCGTGCACAACGAAGGGC (F)
	CCCAGCCGTTGCGGGTCGGGGTTTCCACTTCGGTCAGCAGGCTATACACCGGAGTGAAAGAG (R1)
	CCCAAGCTTCACTAATCGCTGCTATCGCTGCAGCGGCATTCCCAGCCGTTGCGGGTCGGG (R2)
VP1-CP/ VP1 NΔ24CP Adaptor	CATGGTACGGTGACACCAGCACTAACAACGTGAGAGGTGACCTTCAAGTGTTAGCTCAGAAGGCAGAAAGAACTCTGCCTGGGTCGA (F)
	CTAGTCGACCCAGGCAGAGTTCTTTCTGCCCTCTGAGCTAACACTTGAAGGTCACCTCTCACGTTGTTAGTGCTGGTGTCACCGTAC (R)
2C-CP/2C NΔ24CP adaptor	CATGGGAATGGCTGGACAACGCGCGTCAAGCGGGGTCGACGCGTGGGA (F) CTAGTCCCACGCGTCGACCCCGCTTGACGCGCGTTGTCCAGCCATTCC (R)
26G_N_VΔ24CP*	GATACCGGCGAAGTGATTCTGAACAGCTATCGCATTAACCATTATCGCACTCGTGTGGTCCAACC (F1)
	CGCCCATGGGGAGAGTTGAATGCGAAATTGCGCTGAACAAAGATACCGGCGAAGTG (F2)
	CCCAAGCTTCACTAATACACCGGAGTGAAAGAGTCGTCAAACGTAGGTCTGAC (R)
31G_C_VΔ24CP*	GAAGAATGGGGCTGCCTGGCGATTAACGATGGCTGCCTGTATGGCAGCTGCCAGGATATTACTCGTGTGGTCCAACC (F1)
	CGCCCATGGGGTTTAGCAAAGAACGCAGCAGCAACTGGGGCTGCGAAGAATGGGGCTGC (F2)
	CCCAAGCTTCACTAATACACCGGAGTGAAAGAGTCGTCAAACGTAGGTCTGAC (R)
Δ24CP15G_N_*	GGGCCATGGGGACTCGTGTGGTCCAAC (F)
	CTTTCACGCAGCATTCGCTCACATACACCGGAGTG (R1)
	GGGAAGCTTCTAGCTTTTAATAATGCTAATATCATCTTTCACGCAGC (R2)
Δ24CP16G_C_*	GGGCCATGGGGACTCGTGTGGTCCAAC (F)
	CATTCTTCGCAGCCCCAGTTGCTGCTATACACCGGAGTG (R1)
	GGGAAGCTTCTAATCGTTAATCGCCAGGCAGCCCCATTCTTCGCAG (R2)

Restriction enzymes (*Bam*HI, *Hind*III, *Nco*I, *Pst*I, *Spe*I and *Sst*I) and adaptor overhangs are shown in underlined letters.* Epitopes were PCR-fused to the CPs by two rounds of PCR

### CCMV-derived VLPs containing Schmallenberg virus peptide sequences

**Fig. 9:**
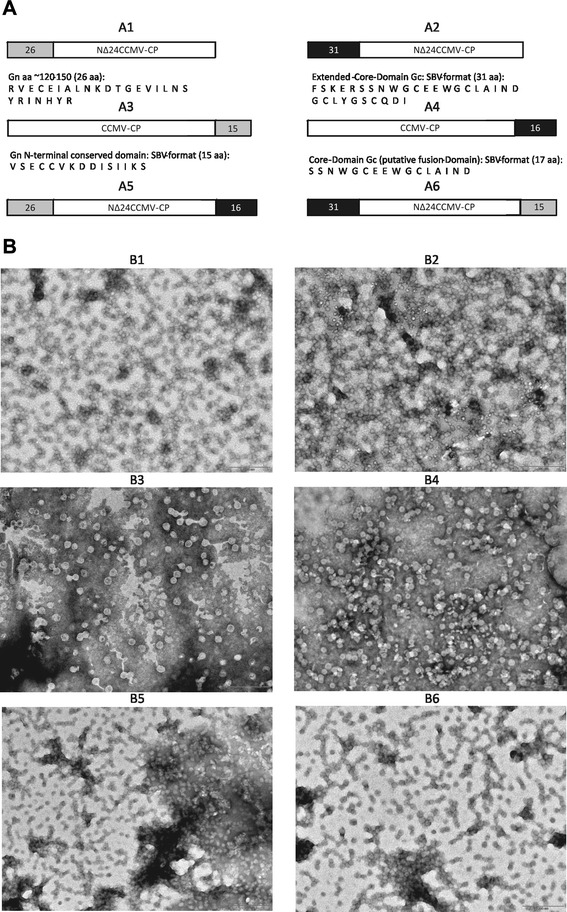
Schematic view and electron micrographs of Schmallenberg virus (SBV) Gn, Gc CCMV-based chimeras. **a** Location and sequence of CCMV constructs harboring SBV glycoprotein peptides fused to N- and C-termini including N-terminal single fusions (*a*1–*a*4) and dual terminal fusions (*a*5, *a*6). **b** Electron micrographs of chimeric CCMV VLPs co-expressing SBV epitopes as depicted in panel a (*b*1–*b*6)

The analysis of CCMV-CP chimera indicated that the extremities of the CCMV CP protein, or the N terminus of a NΔ24-CP mutant could be exploited for epitope fusions in light of subunit vaccine production, whilst maintaining VLP formation. To further substantiate the usefulness and rapid ease of exploitation of CCMV VLPs for the production of potential subunit vaccines, peptide sequences were selected based on a search for conserved sequences or epitope predictions, from the G_N_ and G_C_ glycoproteins of the recently emerged Schmallenberg orthobunyavirus and fused at the aforementioned sites either singly or at both ends simultaneously (Fig. 9a). After expression and VLP formation, as expected, VLPs were readily obtained for all chimera designed (Fig. 9b).

## Discussion

Nowadays numerous virus-like particles, due to their ability to support a strong natural immune response, are being exploited as scaffolds to produce peptide-based vaccines. In this study we have demonstrated the potential of CCMV VLPs as scaffold for foreign epitope presentation, and identified sites for peptide fusions without abolishing VLP formation. While insertions of a 6xHis-tag and M2e (varying in size from 7, 15 to 23 aa) into predicted external loops βB- βC, βD-βE, βF-βG and βH-βI abolish VLP formation, their fusions at the N- and/or C-terminus of CCMV CP or the N-terminus of a mutant lacking the first 24 N-terminal aa residues (NΔ24) still maintain VLP formation. The possibility to further explore these termini for epitope fusion has been supported by VLP formation of CCMV CP chimera containing T- and B cell epitopes from IAV (HA, M2e), and FMDV (2C, VP1). The exploitation of the C-terminus, however, seems limited since fusions of up to 8 aa (His and HA) did not abolish VLP formation, while larger epitopes i.e. M2e(23) gave rise to more amorphous and unstable VLPs. All epitopes fused were detectable with the exception of those from CP-His and 2C-NΔ24-CP, which may have been caused by protein misfolding or the epitope sequence, e.g. in case of 2C, being trimmed in size too much and thereby not being recognized anymore by the antisera. The ease and speed at which additional chimeric VLPs containing SBV epitopes at both termini of CCMV CP have been made underscored the feasibility of CCMV VLPs as scaffold for epitope presentation.

CCMV CP and derivatives expressed in *E. coli* consistently ended up in the insoluble fraction. To circumvent this problem and generate VLPs a protocol has been established that involves a denaturation and resolubilization step, prior to VLP reassembly and subsequent purification by ultracentrifugation on a sucrose cushion. In this way, all CCMV CP chimeras that contained an N-terminal fusion up to 27 aa and ended up in a pellet after sucrose cushion corresponded with the presence of VLPs, with the exception of those containing a relatively large C-terminal fusion. For the latter, the presence of a (MMPC) pellet corresponded either with amorphous aggregates or VLPs.

Abolishment of VLP formation from CCMV CP containing insertions into the 4 selected loops, indicates that these do not tolerate any insertion. Only two hydrophilic loops from these, i.e. βB-βC and βF-βG, are predicted as being exposed and potentially antigenic. While our data did not identify loops that tolerated small epitope insertions, previous work claimed a predicted surface loop in which the insertion of an 11 aa peptide (referred to as CPPep11) still maintained VLP formation. However, the location of the loop has not been described to allow a comparison to the data presented here [25].

Although no VLPs were generated when insertions were made in any of the loops tested, only βF-βG still supported formation of MMPCs when epitopes of varying size were inserted (Fig. 4a_1-4_). The failure to correctly fold into VLPs might be due to loss of the ability to switch between the pentameric and hexameric conformations of the chimeric CP. Earlier studies showed that formation of VLPs from Cowpea mosaic virus (a comovirus with icosahedral particles) containing epitope insertions in the loops (βC’-βC” and βE-αB) depended on both pI value and size of the peptide [28]. In our case we consistently observed more MMPC of CCMV βF-βG-M2e(15) than βF-βG M2e(7) and βF-βG-M2e(23) (Fig. 4a_1-4_) which likewise could indicate that, although VLPs were not generated, composition and peptide size both influence the refolding status of CCMV CP. In bacteriophage Qβ, similar problems were encountered and only by mixing wild-type subunits and chimeric subunits in specific ratios chimeric VLPs were obtained [29]. The latter were likely resulting from lowered sterical hindrances between the subunits that lead to an increased interaction and subsequently supported formation of hybrid VLPs. In light of this, we applied a similar strategy to generate VLPs from epitope insertions into loop βF-βG. To this end, βF-βG-M2e(7) was mixed in two ratios (1:1 and 2:1) with CCMV CP and subjected to VLP reassembly. Preliminary data indicate that VLPs were observed when both were mixed 2:1, but not when mixed in equal ratios (unpublished observation). The presence of the βF-βG-M2e(15) subunit in those VLPs still has to be confirmed.

All N-terminal fusions to CCMV CP/NΔ24-CP, whether His-tag or virus derived epitopes, resulted in the formation of VLPs indistinguishable from those of CCMV CP and from CCMV virions, whereas those from fusions to NΔ24 tended to be a bit more heterogeneous in size (Fig. 4 and Fig. 7). The latter was earlier observed, but more strongly with VLPs from a mutant NΔ34 mutant expressed in *P. pastoris* [25]. Furthermore, comparison of VLPs from His-CP and His-NΔ24-CP revealed less staining in the core of VLPs from His-NΔ24-CP. It is known that the N-terminal end of CCMV CP faces to the interior of CCMV particles where it interacts with the RNA genome. Fusion of epitopes at the N terminus thus is likely not leading to a presentation on the surface of VLPs. For this reason it was interesting to see whether removal of most of the N terminal domain, without abolishing VLP formation or leading to an amorphous VLP suspension, would render relatively stable and homogenous VLPs in which epitopes instead of being internalized would become exposed at the outside. However, several attempts to immuno-gold localize M2e(23) on the surface of NΔ24-CP derived VLPs failed.

During this investigation, the VP1 epitope (27 aa) so far has been the largest fusion made at the N-terminus of wt CCMV CP, without abolishing VLP formation. Considering that NΔ24-CP still rendered VLPs, this would suggest that CCMV CP can be used as a scaffold for peptides at least up to 51 aa, subject it is fused at the N-terminus of NΔ24-CP.

In contrast to all N-terminal fusions, CCMV CP chimera containing C-terminal fusions (6xHis-tag, M2e(23) and HA), especially large ones, ended up in more amorphous and larger VLPs. Those from CP-HA revealed two additional types of particles i.e. opened particles and integrated ones, while preparations from CP-M2e showed to contain the largest particles among all chimeric VLPs from this study (Fig. 7). The latter preparations also revealed unique tubular-like particles. Whether these especially arise due to ionization of carboxyl groups at low pH (5.3-5.7) conditions, which affect the assembly and stability of the particles [30], remains to be investigated. In light of this it is interesting to note that after two weeks for most N-terminal fusion CCMV CP constructs VLPs were still stable, while no VLPs were observed anymore for CP-M2e(23). It cannot be ruled out that, in addition to the relatively large size of M2e(23), the presence of two internal cysteine residues in the epitope sequence has a major influence on VLP formation and stability. Since the N-terminus of CCMV CP is known to interact with the viral RNA genome, the ability of the N-terminus (Fig. 8) within VLPs from different CP-chimera to interact with RNA, e.g. its own mRNA from the *E. coli* extract, was analysed. Earlier, studies have shown that both mRNA and host RNAs from *P. fluorescencs* are encapsulated in CCMV CP VLPs [26]. Here, we observed that bacterially synthesized CP-encoding RNA molecules were similarly detected in VLPs from CCMV CP and CP chimera containing N-terminal fusions of VP1 or 2C, but not in those from M2eNΔ24-CP and HA-NΔ24-CP. Other studies already indicated that VLP-formation is not triggered by RNA/DNA molecules as a treatment with RNaseA and DNase does not abolish the formation of VLPs from CCMV CP [23, 26]. The lack of RNA from the M2eNΔ24-CP and HA-NΔ24-CP derived VLPs is not just due to the N terminal deletion, since VLPs from NΔ24-CP still contained RNA. Instead, the lack of RNA is likely caused by the M2e and HA sequences that provide a negative charge under conditions of VLP reassembly and thereby do not support encapsulation of RNA. This is supported by other studies where VLPs lack RNA in case the (positively charged) N-terminus of CCMV CP is replaced for a negatively charged one [25]. Although the presence of RNA in VLPs from NΔ24-CP, lacking the basic residues from the first 24 aa, was unexpected its remaining N-terminal sequence with neutral to somewhat positive charge still seemed sufficient to support RNA encapsulation. Altogether, the data indicate that epitope fusions at the N-terminus that provide a clear negative charge prevent RNA from becoming encapsulated. From VLPs harbouring a C-terminal fusion of M2e or HA, only those from CP-HA contained RNA. Although speculative, the absence of RNA from VLPs of CP-M2e could be caused by instability of the (amorphous) VLPs, allowing the RNA to become more accessible to nucleases and leading to its degradation.

To ensure that CCMV VLPs, when used as scaffold for epitope presentation or drug delivery nanoparticles, comply with safety rules they should be free from nucleic acids. Chimeric VLPs therefore have to be treated with RNase A and DNase before reassembly [23, 26]. CCMV particles have already been shown feasible for applications in biotechnology such as the encapsulation of anionic non-genomic polymers in CCMV VLPs lacking genomic materials [31]. Recently also DNA origami structures coated with CCMV capsid proteins were efficiently delivered into human cells [32], and viral coat proteins shown to encapsulate gold nanoparticles [33].

While data presented in this study indicate that VLPs from NΔ24-CP present one of the most interesting scaffolds for epitope display, it is highly unlikely that very large sequences can be fused to its N-terminus without disrupting VLP formation. Recently another strategy has been developed [23] that might circumvent this problem. During this strategy a positively charged K-coil sequence (KIAALKE_[3]_) was fused at the N-terminus of CCMV CP. Another, negatively charged E-coil (EIAALEK_[3]_) sequence, able to heterodimerize to the K-coil, was fused to eGFP. Mixing of K-coil-CP in a certain ratio with CCMV CP subunits, enabled the encapsulation of 15 E-coil-eGFP molecules during reassembly of CCMV VLPs. Although this strategy allows encapsulation of very large molecules, due to volume constraints of the core, the number of these will be limited.

Whether (CCMV-derived) VLPs present the best and most widely applicable nanoscaffolds for various purposes, or others like DNA origami structures [34] turn out to be more promising for these in the future remains to be investigated.

## Conclusions

In this study we have demonstrated the feasibility of CCMV-based VLPs as scaffold for epitope presentation, but limited to fusions of peptide sequences to the N and/or C-terminus of CCMV CP or the N-terminus of a mutant lacking the first 24 N-terminal aa residues (NΔ24). Several CCMV CP chimeras have been constructed that resulted in the formation of VLPs containing various (neutralizing) viral epitopes from IAV (HA and M2e), FMDV (VP1 and 2C) and SBV (G_N_ and G_C_), although their real potential in the application for vaccine purposes still has to be demonstrated.

Recently, CCMV particles have been shown to be biocompatible after injection into mice (no observed levels of toxicity and accumulation in specific organs), which underlined their appropriateness and potential for medical applications [24].

## Methods

### Plasmid DNA and cloning

CCMV CP was PCR amplified from a plasmid DNA template (pFBD/CCMV-CP) using Phusion DNA polymerase (Promega) and a primer set to introduce *Nco*I and *Hind*III restriction sites for feasible cloning purposes. Based on the crystal structure of the CCMV CP subunit [19], four different loops were selected to be tested as potential sites for epitope insertion. Insertion of a 6xHis-tag into the predicted loops, including βB-βC, βD-βE, βF-βG and βH-βI (Fig. 1), was performed using PCR mediated mutagenesis, to introduce *Spe*I and *Sst*I restriction sites, and subsequent cloning of a His-tag adaptor into the loops. Constructs containing terminal-end fusions with a His-tag were engineered using a two-step PCR (Fig. 2) during which sequences for the *Nco*I and *Hind*III restriction sites and the 6xHis-tag were provided by the primers (Table 2). Since IAV in human and FMDV in livestocks both cause economically important diseases [35, 36], totally 4 genuine epitopes have been selected. The sequence for the ectodomain of non-glycosylated membrane protein M2 (M2e: SLLTEVETPTRNGWECRCSDSSD) a highly conserved epitope across all influenza A isolates, used for terminal fusions within CCMV CP, was selected based on a consensus amino acid sequence of M2e [37]. M2e has been shown 100 % protection in vaccinated animals against influenza strain H1N1 [13]. For the insertion of size variants of the M2e epitope into the loops of CCMV CP, adapters were designed coding for shorter versions of M2e containing 7 (SLLTEVE) or 15 (SLLTEVETPTRNGWE) aa residues and cloned into the loops. An N-terminal CP mutant, lacking first 24 amino acid residues NΔ24, was generated to abolish interaction with non-cognate host RNA and improve chances of obtaining RNA-free VLPs. To generate an M2e fusion to N∆24-CP, the sequence was subcloned from pDONR201M2eN∆24-CP via *Nco*I and *Hind*III restriction sites (Fig. 2). A T-cell activating epitope of H5N1 subtype in chickens i.e. HA (aa residues 246-260 WTILKPNDAINFESN) was selected from strain A/Hong Kong/156/97. This epitope has a potential use in generation of vaccine in chickens [38]. Additionally two epitopes were selected from FMDV serotype O, the most prevalent serotype worldwide [39]; the first one presenting a B/T-cell epitope from the structural VP1 protein (aa residues 136-160, YGDTSTNNVRGDLQVLAQKAERTLPGS, genbank accession no. CAC22206) [40, 41] and the second one a T-cell epitope from the nonstructural 2C protein (aa residues 68-76, EWLDNARQAGSTR, accession no. AJ539137) [42]. Epitope sequences of HA, VP1, 2C and M2e were PCR-fused to the N or C-terminus of CP (Fig. 5) and cloned into the pET28a (Novagen) expression vector. The integrity of constructs was verified by DNA sequencing. The sequence of all primers and adapters used for the construction of CCMV CP chimera are listed in Table 2.

### Recombinant CCMV CP expression in E. coli

Constructs were transformed into *E. coli* BL21 (DE3). A 100 ml culture containing 50 mg/l kanamycin was freshly inoculated with 1 ml of the overnight culture and grown at 37 °C until an OD_600_ of 0.6 was obtained. Bacteria were induced for protein expression with 0.25 mM IPTG and incubated at 37 °C for another 4 hours. Then, cells were pelleted by centrifugation at 5000 rpm for 15 minutes at 4 °C and stored at -20 °C until further analysis.

### In vitro disassembly and reassembly of CCMV CP to VLPs

CCMV VLPs were re-assembled from insoluble CP aggregates collected from *E. coli* lysates. To this end, a disassembly-reassembly process was carried out entirely at 4 °C, as follows: *E. coli* cells induced and incubated overnight were pelleted and resuspended in 5 ml of disassembly buffer (0.020 M Tris-HCl, 0.9 M NaCl, 0.001 M DTT pH 7.4). Next, cells were lysed by continuous ultra-sonication (Sonics Vibra Cell, CT., USA) during 3 rounds of 1 min at 40 % amplitude (output 6). The lysate was centrifuged at 5.000 rpm for 15 min and the insoluble fraction resuspended in 10 ml of disassembly buffer containing 8 M urea and again sonicated but this time at 20 % amplitude using pulses of 10 seconds until the pellet was entirely dissolved. Thereafter another 15 ml of disassembly buffer was added and mixed with the sonication liquid, the sample was centrifuged at 15,000 rpm for 15 min. The supernatant was collected and dialyzed 6 times against dialysis buffer (0.01 M Tris-HCl pH7.4). The soluble CCMV CP fraction was finally reassembled into VLPs during 2 dialysis steps against reassembly buffer (0.1 M NaAc, 0.9 M NaCl, 0.010 M MgCl_2_ pH 4.8) and concentrated by ultracentrifugation (Beckman) on a 30 % sucrose cushion at 40,000 rpm for 3 hours. The pellet was resuspended in 1-2 ml of virus buffer (0.1 M NaAc, 0.001 M EDTA, pH 4.8) and stored at 4 °C until further analysis.

### SDS-PAGE and immunoblot analysis

Bacterial cell lysates containing expressed (chimeric) CCMV CP protein or samples of purified VLPs were resolved onto a 15 % SDS-PAGE gel. Proteins were transferred onto Immobilon P membrane (Millipore Corp., MA, USA) and screened with anti-CCMV polyclonal crude antiserum (1:1000), anti-His (1:1000) and anti-M2e monoclonal antisera (1:1000 of a 1 mg/ml stock; Abcam Inc., Cambridge, MA). For VP1 and 2C epitopes, a serum from FMDV-infected guinea peg (kindly provided by Dr. A. Dekker, Central Veterinary Institute, Lelystad, the Netherlands) was used (1:500 crude serum).

### EM and immuno-electron microscopy

The reassembly of all CCMV CP proteins into VLPs was verified by transmission electron microscopy. To this end, protein samples collected after *in vitro* reassembly and sucrose cushion were diluted 10x in virus buffer and 10 μl placed on 400 mesh copper grids (AURION, Wageningen, The Netherlands) for 2 min followed by negative staining. For immuno-electron microscopy [43] 10 μl of the samples was placed on 400 mesh nickel grids (AURION) for 5 min and followed by blocking using virus buffer containing 1 % BSA for 20 min. Polyclonal (anti-CCMV) and monoclonal (anti-His and anti-M2e) antisera were used (1:1000) as primary antibodies and goat-anti-rabbit- and anti-mouse-gold (9 nm) conjugates (AURION) used at a 1:20 dilution in virus buffer (supplemented with 1 % BSA) as secondary antibodies. After 6 washing steps, samples on the grids were fixed using virus buffer containing 1 % glutaraldehyde. The preparations were negatively stained with 2 % Uranyl acetate (pH 3.7) for 1 min and dried at room temperature. The grids were examined with a JEOL JEM-1011 transmission electron microscope using wild type CCMV CP derived VLPs as a positive control.

### Detection of RNA in purified VLPs

A major safety issue related to the exploitation of VLP-based systems for vaccine purposes deals with the presence/absence of genetic material within VLPs. To analyze the presence of mRNA transcribed from the pET-28-CCMV CP-chimera plasmids and encapsulated in VLPs, 200 μl purified VLP suspensions from CCMV CP, NΔ24-CP, M2e(23)NΔ24-CP, HA-NΔ24-CP, VP1-NΔ24-CP, 2C-NΔ24-CP, VP1-CP, 2C-CP, CP-HA and CP-M2e(23) was subjected to a total RNA isolation protocol using Trizol according to the manufacturer’s instructions (Invitrogen). As a negative control, a sample of IPTG-induced BL21 cells containing empty pET-28 plasmid DNA was included. To detect CCMV-CP-specific RNA molecules transcribed from pET28, RT-PCR was performed on total RNA purified from VLPs using specific primer sets (Table 2).

Charges of CP and CP-chimera were calculated using Protein Calculator v3.3, (C. Putnam, The Scripps Research Institute, USA) at pH 7.4 and 4.8, corresponding to buffer conditions reflecting the CCMV CP disassembly and CCMV VLP reassembly steps as described above.

## Abbreviations

CCMV: Cowpea chlorotic mottle virus
VLPs: Virus-like particles
CP: Coat Protein
IAV: Influenza A virus
M2e: Ectodomain membrane protein M2
HA: Haemagglutinin
FMDV: Foot and Mouth Disease Virus
N-terminal (end): Amino- terminal
C-terminal (end): Carboxyl-terminal
aa: Amino acid
N∆24-CP: N-terminal 24 aa deletion mutant
*i*: ionic strength
SDS-PAGE: Sodium Dodecylsulfate-Polyacrylamide Gel Electrophoresis
MMPCs: Macro molecular Protein Complexes
EM: Electron microscopy
pI: Isoelectric point
NΔ34: N-terminal 34 aa deletion mutant
K-coil: KIAALKE heptad positively charged sequence
E-coil: EIAALEK heptad negatively charged sequence
pFBD/CCMV-CP: pFastBac plasmid containing CCMV-CP
pDONR201M2eN∆24-CP: a Gateway donor vector containing N-terminal 24 aa deletion mutant
*E. coli* BL21 (DE3): cells carry the lambda DE3 lysogen for overexpression of the protein in *E. Coli*
IPTG: Isopropyl β-D-1-thiogalactopyranoside
DTT: Dithiothreitol
BSA: Bovine Serum Albumine
